# Lane Kink

**Published:** 1915-01

**Authors:** 


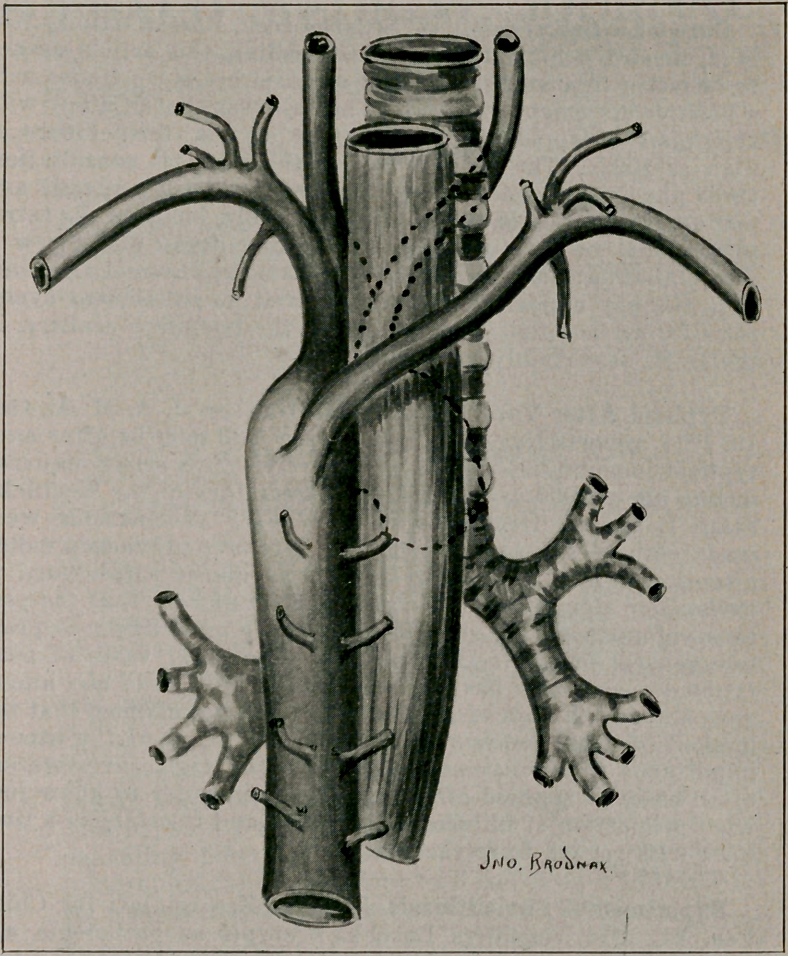

**Lane Kink. Wm.** Francis Campbell of Brooklyn, "Med. Times," December, 1914, contends that every case of this condition examined has been found associated with incompetent ileo-caecal valve. He therefore considers it a compensatory condition, although the explanation of how the kink is developed to prevent reflux into the ileum, is not obvious. Naturally, if this is the correct conception, the condition should not be operated upon, unless with the object of restoring the competency of the valve.

